# Neuroimmune Interactions in Chronic Pain and Neuroinflammation: Peripheral Nerve Injury, Neuroimmune Mechanisms, and Therapeutic Recovery

**DOI:** 10.3390/biom16101410

**Published:** 2026-09-28

**Authors:** Ahmed I. Anwar, Lillian G. Jenkins, Benjamin J. Branch, Gabrielle A. Cassagne, Gavin M. Daigle, Marc J. Levin, Osman Yaray, Simeon D. Crosby, Alan D. Kaye

**Affiliations:** 1 School of Medicine, Louisiana State University Health Sciences Center at Shreveport, 1501 Kings Highway, Shreveport, LA 71103, USA; 2 Department of Chemical and Biological Engineering, Colorado School of Mines, Golden, CO 80401, USA; 3 Department of Family Medicine, LSU Health Shreveport, Shreveport, LA 71103, USA; 4 College of Science, University of Arizona, Tucson, AZ 85721, USA; simcrosll@arizona.edu; 5 Department of Anesthesiology, Louisiana State University Health Shreveport, 1501 Kings Highway, Shreveport, LA 71103, USA

**Keywords:** pain, neuro-immune, chronic pain, inflammation, nerve damage, central sensitization, peripheral sensitization, damage-associated molecular patterns, cytokines

## Abstract

The pathogenesis of chronic pain includes persistent neuroimmune interactions that contribute to both peripheral and central pain sensitization. Tissue injury and peripheral nerve injury activate immune and glial pathways through various cytokines, chemokines, complement, damage-associated molecular patterns (DAMPs), and neuroimmune feedback loops involving nociceptors, macrophages, mast cells, microglia, and astrocytes. This review examines the mechanisms by which these interactions contribute to persistent nerve injury, neuroinflammation, and the transition from acute injury to chronic pain. Biomarkers and their molecular targets may improve identification of the different pain mechanisms involved while also supporting immune-based therapeutic strategies, although their clinical utility and consistency remain limited. Current and new immune-based therapies that target cytokine signaling, glial activation, complement pathways, neurotrophic factors, and inflammatory resolution mechanisms have demonstrated promising effects in preclinical studies. Resolution of neuroinflammation also involves immune regulation, cellular reprogramming, and peripheral nerve regeneration. There is a unique role for neuroimmune signaling in chronic pain, and the current literature suggests that targeting persistent inflammatory pathways promotes resolution and nerve repair. Overall, this review aims to highlight the potential approaches for recovery and the role inflammation plays in chronic pain states.

## 1. Introduction

### 1.1. Overview of Chronic Pain

Chronic pain is a complex condition that differs from acute pain in both duration and clinical course. Acute pain generally serves as a protective response to injury and improves as the underlying condition heals. Chronic pain, in contrast, persists or returns beyond the expected period of healing and is generally defined as lasting three months or longer [1,2]. In this regard, the International Association for the Study of Pain (IASP) defines pain as an unpleasant sensory and emotional experience associated with, or resembling, that associated with actual or potential tissue damage [3]. This definition recognizes that pain is not simply a measure of tissue injury and can be influenced by a variety of biological and psychosocial factors [4].

The International Classification of Diseases, 11th Revision (ICD-11), further divides chronic pain into chronic primary pain and chronic secondary pain. Chronic primary pain refers to pain that is the main health condition itself, while chronic secondary pain occurs in association with an underlying disease or condition [3,5]. Thus, this classification reflects the wide range of conditions that can lead to chronic pain and the fact that its underlying mechanisms can vary considerably between patients.

Chronic pain affects a substantial portion of the population worldwide. Estimates vary from approximately 11% to 40%, depending on the population studied and the definition used [1,4]. In the United States, an estimated 51.6 million adults (20.9%) experienced chronic pain in 2021, including 17.1 million (6.9%) who had high-impact chronic pain that significantly interfered with daily activities [6]. Low back pain, headache disorders, osteoarthritis, and neck pain are also among the leading causes of years lived with disability worldwide [7].

The effects of chronic pain extend well beyond the pain itself. In the United States, the combined costs of medical care and lost productivity were estimated at $560–635 billion annually in 2010, which was greater than the yearly costs associated with cancer, heart disease, and diabetes [4,8]. A more recent analysis estimated the total cost of chronic pain at $722.8 billion in 2021 [9]. Chronic pain also affects some populations more than others, including women, individuals with lower socioeconomic status, rural residents, and veterans [4,6]. In addition to physical limitations, chronic pain is associated with depression, increased suicide risk, and substance use, adding to its effects on both patients and the healthcare system [6].

Despite how common and costly it is, chronic pain remains challenging to treat. Medications such as opioids, anticonvulsants, and antidepressants primarily affect neuronal signaling, but many patients do not experience adequate or lasting relief. Fewer than 30% of patients achieve meaningful improvement with commonly used pharmacologic treatments [10], and response rates for medications used to treat neuropathic pain remain below 50% [11,12]. Long-term treatment can also be limited by adverse effects and safety concerns, particularly with opioid therapy [13]. These limitations make it important to better understand the mechanisms that allow pain to persist and to identify new treatment targets.

### 1.2. Neuroinflammation in Chronic Pain

Neuroinflammation is an important contributor to the development and persistence of chronic pain. It involves an inflammatory response within the nervous system that includes activation of glial cells, particularly microglia and astrocytes, as well as the release of inflammatory cytokines and chemokines [14]. Peripheral immune cells can also be recruited to areas of nerve injury, and changes in vascular permeability may further contribute to the inflammatory response [14]. Under normal circumstances, these responses are carefully regulated and help protect injured tissue and promote healing. Problems can arise when the inflammatory response continues for too long, allowing processes that are normally protective to contribute to ongoing pain.

Inflammation following tissue or nerve injury is usually temporary. It helps clear damaged tissue, initiate repair, and support healing before eventually resolving. In some cases, however, this process does not completely shut down after the original injury has healed. Persistent inflammation can then contribute to disease rather than recovery [10,15]. Chronic pain may therefore result from impaired mechanisms responsible for resolving inflammation. Interestingly, attempts to suppress inflammation too early with medications such as nonsteroidal anti-inflammatory drugs (NSAIDs) or corticosteroids may interfere with some of these natural resolution pathways and potentially contribute to persistent pain [10,12].

Ongoing neuroinflammation can affect pain signaling in both the peripheral nervous system (PNS) and the central nervous system (CNS). After tissue or nerve injury, inflammatory mediators activate sensory neurons and nearby glial cells, increasing the sensitivity of peripheral nociceptors in a process known as peripheral sensitization [14,16]. Continued activation of glial cells and release of cytokines can also increase the responsiveness of neurons in the spinal cord, contributing to central sensitization [14]. These changes can make the nervous system more responsive to painful stimuli and may contribute to the transition from acute pain to chronic and more widespread pain [14].

### 1.3. Neuroimmune Crosstalk in Pain Pathophysiology

Changes within the nervous system alone do not fully explain why chronic pain develops and persists. The nervous and immune systems communicate closely with one another, and this interaction appears to play an important role in chronic pain [17]. Following injury, neurons and glial cells release signaling molecules that activate nearby immune cells and recruit additional immune cells from the circulation. These immune cells then release cytokines and chemokines that can affect pain signaling at several levels, including peripheral nerve endings, along the nerve, within the dorsal root ganglion (DRG), and at synapses in the CNS [16].

In the PNS, macrophages, T lymphocytes, and mast cells can contribute to nociceptor sensitization through the release of inflammatory mediators such as interleukin-1β (IL-1β), interleukin-6 (IL-6), and tumor necrosis factor-α (TNF-α) [10,15]. These interactions increase the sensitivity of nociceptors and can help maintain pain signaling. In the CNS, the activation of microglia and astrocytes contributes to neuroinflammation and can lead to changes in synaptic signaling, increased excitatory neurotransmission, and reduced descending inhibition of pain [14].

Importantly, the immune system does not always have a pain-promoting role. Depending on the cells and pathways involved, immune responses can either contribute to pain or its resolution. Pro-inflammatory responses involving Th1 and Th17 cells can promote development and persistence of pain, while Th2 cells, regulatory T cells, and macrophages can contribute to pain resolution and to recovery [10,18]. The relationship between the immune and nervous systems is therefore more complex than simply being a source of inflammation. These interactions can contribute to both persistence of chronic pain and processes involved in recovery.

### 1.4. Clinical Significance and Rationale for Review

Understanding the role of neuroimmune interactions in chronic pain is clinically important because current treatments do not provide lasting relief for many patients. Most conventional analgesics primarily target neuronal signaling, while chronic pain involves changes in both the nervous and immune systems. This has led to growing interest in treatments that target immune pathways involved in pain. Early studies of repurposed immunomodulatory therapies, including anti-TNF agents, colony-stimulating factor-1 receptor (CSF-1R) inhibitors, and pro-resolving mediators, suggest that targeting these pathways may affect pain outcomes [13]. However, results have been variable, and there are still important questions regarding safety, treatment response, and how well these therapies will translate into clinical practice.

Another challenge is the lack of reliable biomarkers that can identify mechanisms contributing to an individual’s pain. Although biomarkers could potentially improve diagnosis, prognosis, and treatment selection, there is currently no fully validated clinical biomarker for chronic pain [19,20]. Research involving cytokine profiling, proteomics, metabolomics, neuroimaging, and quantitative sensory testing is beginning to show differences between patients and may help identify specific immune-related pain profiles, or endotypes [13,21]. These approaches could eventually help move chronic pain treatment away from a trial-and-error approach and toward treatments that are better matched to the underlying mechanisms of pain in individual patients.

### 1.5. Purpose of This Review

As a narrative review, the present investigation examines current evidence on the role of neuroimmune interactions in chronic pain, with a particular focus on immune-mediated peripheral nerve injury and neuroinflammation. It first discusses neuroimmune mechanisms involved in development and persistence of chronic pain. The review then examines how immune-mediated peripheral nerve injury contributes to neuroinflammation and to ongoing pain.

The following sections also address biomarkers and molecular targets that may help identify specific pain mechanisms and guide immune-based treatments. Current and emerging immunomodulatory therapies are reviewed, along with challenges that have limited translation into clinical practice. Finally, this review discusses future directions for the field, including potential for biomarker-guided and personalized approaches to chronic pain treatment. Although the primary focus of this review is on chronic and neuropathic pain, selected evidence from other inflammatory, immune-mediated, and tissue-injury conditions are discussed when it provides relevant mechanistic or translational insight into the neuroimmune processes. Importantly, this review distinguishes between mechanistic plausibility, preclinical evidence, and demonstrated clinical efficacy, recognizing that promising neuroimmune targets have not consistently translated into effective clinical treatments.

## 2. Methodology

This present investigation utilized the PubMed database to identify the relevant literature for this narrative review that examined mechanisms, pathophysiology, biomarkers, and management strategies of chronic and neuropathic pain, with an emphasis on neuroimmune interactions. The search was conducted in August 2026, using relevant keywords and Boolean combinations of key terms and related terms such as “chronic pain”, “neuropathic pain”, “neuroinflammation”, “neuroimmune interactions”, “microglia”, “astrocytes”, “macrophages”, “cytokines”, “chemokines “and “pain resolution”, as well as related terms including “immune cells” and “biomarkers”. No strict data restrictions were applied. Retrieved studies were screened for relevance to the scope and specific themes of this narrative review, including the interaction between inflammation and pain, neuroimmune interactions underlying pain, and the roles of microglia, astrocytes, and other relevant mechanistic, clinical, biomarker, or therapeutic evidence addressing these themes. Reference lists from selected articles were also manually reviewed to identify additional research, and priority was given to peer-reviewed systematic reviews, meta-analyses, clinical studies, and mechanistic studies. Evidence was prioritized based on study design, relevance to human pain mechanisms, and potential therapeutic translations. As a narrative review, the literature-selection process was used to identify and synthesize key evidence relevant to the objectives and thematic scope of this review.

## 3. Neuroimmune Mechanisms of Chronic Pain

### 3.1. Peripheral Immune Activation Following Tissue and Nerve Injury

Following cellular damage caused by tissue and nerve injury, dying cells release damage-associated molecular patterns (DAMPs) [22]. DAMPs are endogenous cellular molecules that function as an alarm signal which alerts the immune system to tissue damage [22]. Examples of DAMPs that are released following cellular damage include adenosine triphosphate (ATP), high-mobility group box 1 (HMGB1), heat-shock proteins and cellular debris such as nucleic acid fragments and histones [22].

HMGB1 is widely regarded as the prototypical DAMP, and ATP is best described as a ubiquitous and highly efficient danger signal [23]. Importantly, tissue and nerve injury does not just damage neurons or cells in the local injury site; it rapidly generates danger signals and inflammatory mediators that can initiate innate immune responses [24].

These DAMPs activate pattern-recognition receptors (PRRs) which include TLR (e.g., TLR2 and TLR4 at the cell surface and endosomal TLR3 and TLR7–9), the nucleotide-binding domain, leucine-rich-containing family, pyrin domain-containing-3 (NLRP3) inflammasome, purinergic P2X and P2Y receptors, and the receptor for advanced glycation end products (RAGE), which drive innate immune signaling through the nuclear factor-κB (NF-κB) and mitogen-activated protein kinase (MAPK) pathways [25].

ATP then signals through P2X and P2Y receptors, which promotes the NLRP3 inflammasome to release IL-1β [24]. Inflammatory mediators produced by these pathways include TNF-α, IL-1β, IL-6, chemokines, and prostaglandins, which subsequently go on to act on nearby nociceptors [16].

Peripheral sensitization then occurs, where nociceptors become more excitable and their activation threshold decreases so that normally innocuous stimuli become painful ones [26]. Prostaglandins and cytokines phosphorylate and sensitize transient receptor potential channels (e.g., transient receptor potential vanilloid 1 (TRPV1) and transient receptor potential ankyrin 1 (TRPA1)) and voltage-gated sodium channels (e.g., Na_v_1.7, Na_v_1.8, and Na_v_1.9), which provides the molecular basis for the reduced threshold of these nociceptors. TNF-α acts in part by increasing nociceptor susceptibility to prostaglandin E_2_ via the tumor necrosis factor receptor 1 (TNFR1) [16].

Injured and hyperactive nociceptors release neuropeptides substance P and calcitonin gene-related peptide (CGRP), which promote vasodilation, increased vascular permeability, and immune cell recruitment through a process known as neurogenic inflammation [26]. Substance P induces monocytes and macrophages to release interleukin-1 (IL-1), TNF, and IL-6, while recruited immune cells release additional inflammatory mediators that act on nociceptors [26].

This process creates a relationship in which neurons activate immune cells, and the immune cells in turn activate and sensitize neurons in a positive-feedback loop that promotes the persistence of pain and inflammation following injury. The role of substance P is primarily pro-inflammatory, whereas CGRP has anti-inflammatory effects on myeloid cells by reducing pro-inflammatory cytokine production and increasing interleukin-10 (IL-10) [26].

Persistent inflammation with antigen presentation and sustained cytokine signaling recruits T lymphocytes to the site of injury, which activates the adaptive immune system [26]. The outcome of this process depends on the type of T cell recruited to the location. CD4+ T helper 1 (Th1) and Th17 cells promote inflammation whereas Th2 cells and regulatory T cells (T-regs) suppress pain [27]. Forkhead box P3 (FOXP3) + T-regs suppress neuropathic pain mainly by inhibiting the local Th1 response through the secretion of IL-10 [27].

Depletion of these cell types impairs and delays pain resolution [27,28]. CD8+ T cells mostly function as pain-resolving rather than pro-inflammatory and are required for the resolution of chemotherapy-induced neuropathic pain and the reversal of nerve fiber loss [29,30].

**Figure 1:**
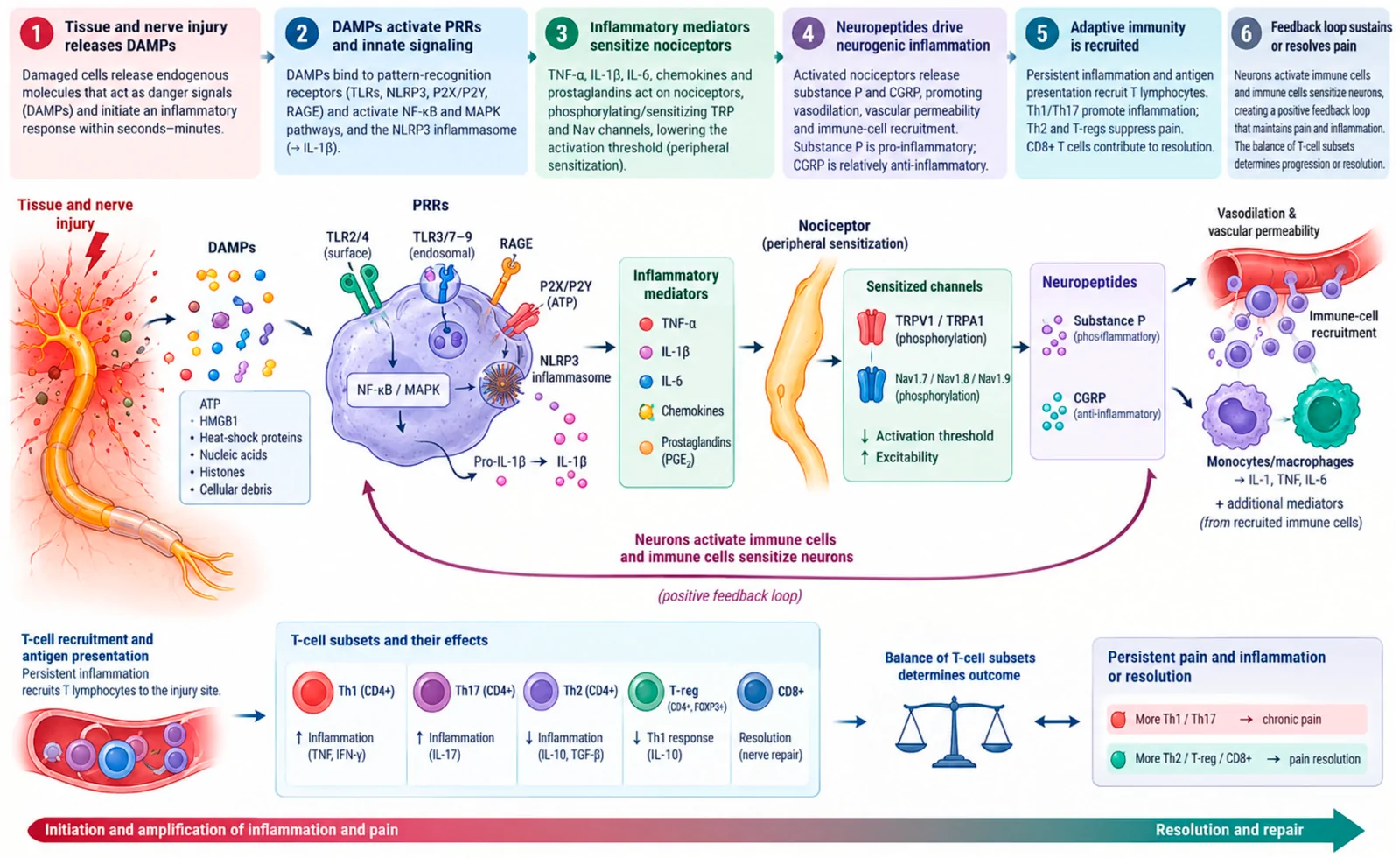
Peripheral immune activation following tissue and nerve injury. Tissue injury releases DAMPs that activate innate immune signaling and inflammatory mediator production. This figure was created using Open AI Chat GPT (GPT-5) with a prompt describing the mechanisms of peripheral nerve injury. The AI generation of this figure is disclosed, and the figure was reviewed and verified for its scientific content.

Once peripheral nerve injury initiates this inflammatory response, CD4+, CD8+, and T-reg subsets can each either promote or restrain the process, making them important mediators of both the progression of pain and its eventual resolution [31]. A schematic of the immune process following injury is seen in Figure 1.

### 3.2. Peripheral Immune Cells Involved in Pain Modulation

After initial injury, monocytes are recruited to the injured tissue or nerve, where they differentiate into macrophages that respond to the local inflammatory environment [32]. The M1-like phenotype of macrophages is pro-inflammatory and produces TNF-α, IL-1β, IL-6, and reactive oxygen species (ROS) [32].

These mediators lower the activation threshold of nociceptors, sensitizing them, which contributes to persistent neuropathic and inflammatory pain at the site of injury [32,33]. In contrast, the M2-like phenotype is reparative and promotes the resolution of injury. These M2 macrophages repair tissue and clear cellular debris by phagocytosis and secrete anti-inflammatory mediators such as IL-10 [32].

M1 and M2 macrophage phenotypes exist along a spectrum and are not fixed categories; thus, a given macrophage’s functionality at the site of tissue damage can shift over time as injury evolves from its initial phase toward resolution [33]. Ultimately, inflammatory and reparative macrophage activity influences whether the pain from the initial injury resolves completely or becomes a chronic problem [33].

Neutrophils are the first immune cells recruited after injury and are initially attracted to the site of cellular damage by chemokines and other inflammatory signals [34]. These neutrophils then migrate through the vascular endothelium and accumulate within the site of damaged tissue where they help initiate the early inflammatory response [34]. Once present within the damaged tissue, neutrophils release cytokines, chemokines, proteases, ROS, and lipid mediators [34].

The factors collectively sensitize nociceptors, contributing to peripheral sensitization [35]. As the dominant immune cell of the early inflammatory phase, neutrophils help initiate the inflammatory response and amplify acute pain and sensitization [33]. These effects are subsequently reinforced by the recruitment of monocytes and macrophages [33,34].

CD4+ T cells that have been recruited to the site of injury help coordinate the immune response, and their effect on pain depends largely on their phenotype [27]. Th1 and Th17 cells are pro-inflammatory subsets that produce cytokines which promote inflammation and pain at the injury site [27,33]. In contrast, Th2 cells produce anti-inflammatory mediators that reduce pain hypersensitivity at the site of injury [27].

CD8+ T cells can also contribute to pain modulation after nerve injury when they interact with injured peripheral nerves and DRG [33]. T-regs play an important role in preventing excessive immune activation [27]. At sites of nerve injury, FOXP3+ T-reg cells release IL-10, which helps suppress the local Th1 response and promotes macrophages to shift toward an M2 phenotype which is more supportive of tissue repair [27]. Depletion of these T-reg cells will increase mechanical hypersensitivity and delay recovery. This suggests that T-reg cells play an important role in resolving pain and that impaired T-reg activity may contribute to ongoing inflammation and chronic pain [33].

Mast cells also play an important role in neurogenic inflammation. Within tissues, mast cells are located near peripheral nerve fibers and are rapidly activated in response to injury or inflammatory signals, including neuropeptides released from sensitized nerve endings [36]. Once the mast cells are activated, they release histamine, tryptase, cytokines, and other inflammatory mediators that increase nociceptor sensitivity and enhance vascular permeability at the site of injury [36].

Nociceptors within the tissue then release neuropeptides such as substance P that activate more mast cells, in part through the mast-cell-specific mas-related G protein-coupled receptor member X2 (MRGPRX2) and mas-related G protein-coupled receptor member B2 (MRGPRB2), creating a self-amplifying positive-feedback neuroimmune loop that may drive the transition from acute to chronic pain [37,38].

**Figure 2:**
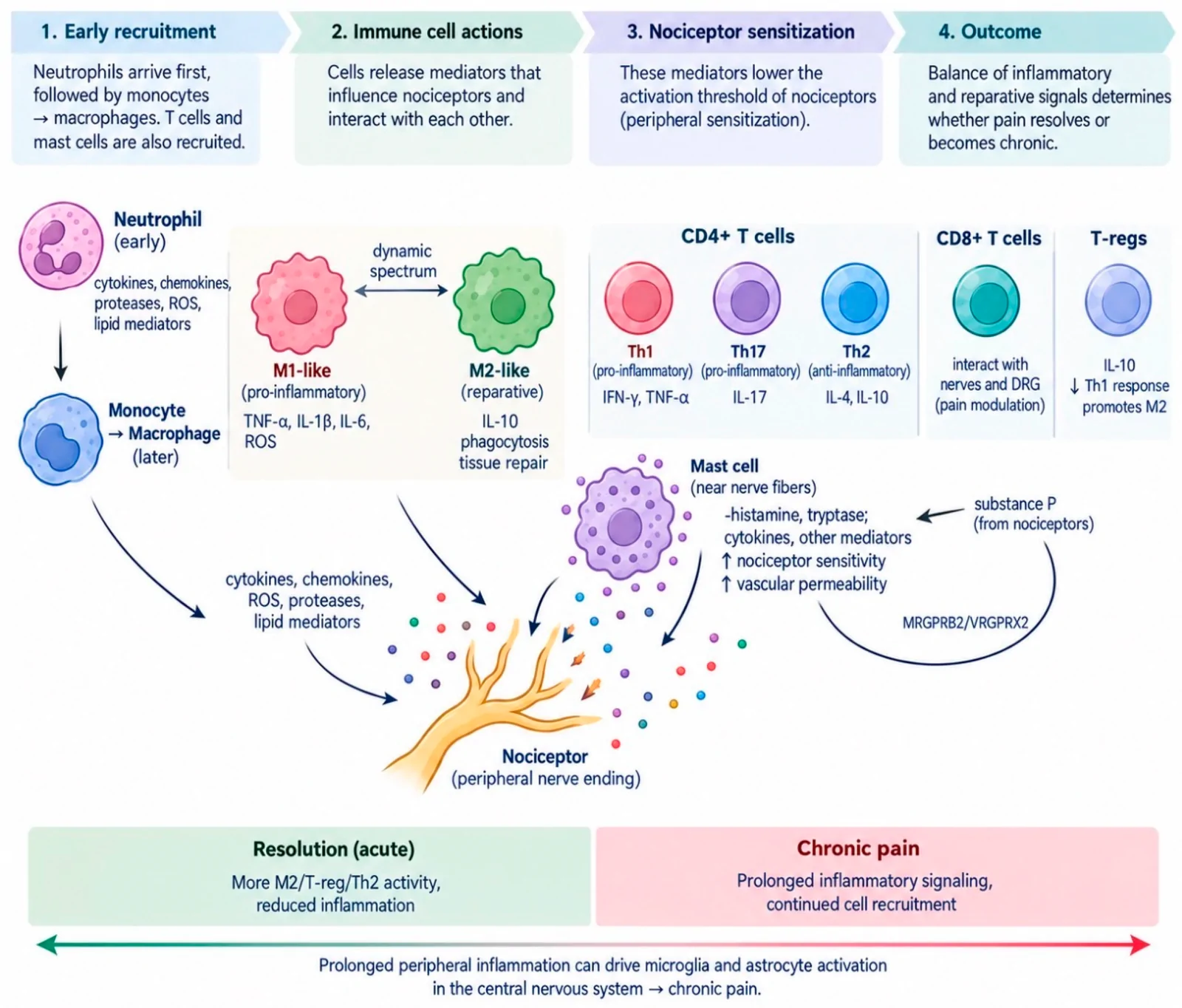
Peripheral immune-cell regulation of pain following tissue or nerve injury. Neutrophils are recruited early after injury, followed by monocyte-derived cells. The balance and persistence of these immune–neural interactions, including signaling mediated by interferon gamma (IFN-γ) and reactive oxygen species (ROS), influence whether pain resolves or progresses toward chronic pain. This figure was created using Open AI Chat GPT (GPT-5) with a prompt describing the mechanisms of peripheral immune cells. The AI generation of this figure is disclosed, and the figure was reviewed and verified for its scientific content.

The interactions among macrophages, neutrophils, T cells, and mast cells and between these immune cells and sensory neurons following injury can play a major role in whether an injury completely resolves transitions to chronic pain [33]. The timing of these interactions as well as the phenotypes of the cells involved also play a major role in the outcome of injury [33]. When this peripheral inflammatory signaling is prolonged, these signals can drive changes within the CNS, where microglia and astrocytes become increasingly important mediators of chronic pain [14]. A schematic of peripheral immune cells and pain modulation is seen in Figure 2.

### 3.3. Central Neuroimmune Responses and Glial Activation

Chronic peripheral nerve injury does not always remain confined to the injured nerve. Prolonged nociceptive input affects the CNS, where it activates glial cells, principally microglia and astrocytes, which become active participants in pain processing [39].

Microglia are the resident immune cells of the CNS [39,40]. Peripheral nerve injury activates microglia in pain-processing regions of the spinal cord, particularly the dorsal horn, where ATP, colony-stimulating factor-1 (CSF1), chemokines (e.g., CCL2 and CX3CL1), and proteases released from the central terminals of injured hyperactive primary afferents bind their microglial receptors [39,40]. Because of this, microglia undergo morphological and transcriptional changes including cell-body enlargement, upregulation of ionized calcium-binding adapter molecule 1 (IBA-1) and CD11b expression, and activation of intracellular cascades such as p38 MAPK [40]. Activated microglial cells release cytokines (e.g., TNF-α, IL-1β, and IL-6), chemokines, and neurotrophic factors like brain-derived neurotrophic factor (BDNF), prostaglandin E_2_ and ATP [39].

All of these factors act on the surrounding dorsal horn neurons within the spinal cord to increase the excitatory synaptic transmissions and decrease inhibitory neurotransmitters (e.g., GABA and glycine), which raises neuronal excitability, producing central sensitization [39]. Once this central sensitization has developed, pain can continue to persist even after the peripheral injury has improved [14]. Microglia play important roles in the clearance of cellular debris, tissue remodeling, repair and injury resolution. Like other immune cells discussed, their function can change depending on timing and their environment [39].

Astrocytes are another major cell type within the CNS that contributes to chronic pain. During long-term inflammation dorsal horn astrocytes secrete chemokines (e.g., CCL2 and CXCL1) and cytokines (e.g., IL-1β, IL-6, interleukin-17 (IL-17), and TNF-α) [41]. These chemokines and cytokines promote central sensitization by facilitating NMDA-receptor-dependent ERK activation and long-term potentiation while disinhibiting nociceptive neurons [39,41]. Besides releasing these inflammatory mediators, astrocytes alter neurotransmitter homeostasis through sustained downregulation of the glutamate transporter 1 (GLT1), which causes glutamate spillover into surrounding tissues and the activation of extrasynaptic NMDA receptors [41].

Astrocyte reactivity is often maintained longer than the initiating injury, which may allow astrocytes to prolong rather than initiate chronic pain [41]. Continued communication between the astrocytes and neurons drives central sensitization, and together, these findings describe a continuous self-reinforcing cascade [14,41]. In summary, these mechanisms provide an overview of how tissues and nerve injury can initiate signaling and chronic pain. The specific mechanisms of peripheral nerve injury and associated inflammatory pathways are examined in greater detail in Section 4. These cells then engage adaptive immune responses through T cell involvement [27].

Inflammatory mediators that are released as a result of these sensitized peripheral nociceptors, and the ensuing persistent nociceptive signaling drives microglial activation in the spinal dorsal horn, followed by astrocyte activation that further prolongs the response. Together, glial–neuronal interactions shift the dorsal horn toward a state of central sensitization that ultimately manifests as chronic pain [19].

## 4. Immune-Mediated Peripheral Nerve Injury and Neuroinflammation

### 4.1. Mechanisms of Peripheral Nerve Damage

Building on the broader neuroimmune mechanisms highlighted in Section 3, this section focuses specifically on the mechanisms of peripheral nerve injury and the associated inflammatory pathways that contribute to neuropathic pain. Following peripheral nerve injury, a series of pathological changes occur that disrupt the normal structure and function of the nerve. The main mechanisms are axonal degeneration, demyelination, and disruption of the blood–nerve barrier (BNB). All these changes impair nerve conduction and cause abnormal pain signaling [42].

Axonal degeneration is one of the earliest responses to nerve injury, most commonly by Wallerian degeneration. When a nerve transection or severe compression occurs, the distal portion of the axon begins to fragment, followed by degeneration of the surrounding myelin sheath [42]. Schwann cells and macrophages together remove axonal and myelin debris to create a favorable environment for axonal regeneration [43]. According to Schenone et al. (2025), in Wallerian degeneration, axonal pathology also includes axonal atrophy and dystrophy [44]. These mechanisms have been extensively studied in traumatic nerve injuries, but remain less well understood in inherited and metabolic neuropathies [44].

The activated Schwann cells promote remyelination and tissue repair; but ongoing inflammation may interfere with recovery and result in continuing demyelination [43,44]. Another important structure is the BNB in peripheral nerve injury [45]. This structure is composed of endoneurial endothelial cells and perineurial cell layers [45]. The BNB regulates the exchange of substances between the blood stream and the endoneurial environment and protects the peripheral nerves [45].

Any damage to this barrier will let the circulating immune cells and serum proteins enter the nerve tissue and intensify local inflammation and tissue injury [45]. The disruption of the BNB is common in traumatic, metabolic, and immune-mediated neuropathies and may represent an attractive therapeutic target [45]. The progress of inflammation will cause reduced conduction velocity, ectopic neuronal firing, and increased nociceptor sensitivity, which contributes to the development of neuropathic pain [43,44].

### 4.2. Cytokine and Chemokine Networks in Pain Development

Inflammatory cytokines play an important role between tissue injury and changes in pain signaling process [46]. TNF-α starts inflammatory cascades which lower the activation threshold of primary sensory neurons and enhance synaptic transmission in the dorsal horn [46]. These processes increase both peripheral and central sensitization [46]. IL-1β can directly activate nociceptors and amplify local inflammatory responses. IL-6 changes nociceptive processing and neuronal plasticity by trans-signaling pathways which contribute to persistent pain [33,46].

Recent studies have provided additional details about the role of IL-6, demonstrating that it stimulates new protein synthesis through the MNK-eIF4E signaling pathway, which regulates protein production in human DRG nociceptors, which are pain-sensing neurons located in the DRG. This may help explain the molecular mechanisms underlying prolonged peripheral sensitization [47]. Yu et al. (2024) demonstrated that a higher level of IL-6 in cerebrospinal fluid increases pain by signal transducer and activator of transcription 3 (STAT3)-mediated activation of astrocytes, highlighting the importance of IL-6 in both peripheral and central pain mechanisms [48].

Chemokines intensify these inflammatory responses by regulating movements of the immune cells and communication between neurons and glial cells [49]. The CCL2 (MCP-1)/CCR2 signaling pathway enhances the recruitment of monocytes and macrophages to injured peripheral nerves and the DRG, thereby sustaining neuroinflammation [49].

Activated spinal astrocytes produce CXCL1 and act on CXCR2 to increase NMDA receptor activity and cyclooxygenase-2 (COX-2) expression, which increases neuronal excitability [49]. CX3CL1 (fractalkine) is expressed on neuronal membranes and interacts with microglia via CX3CR1. Fractalkine becomes soluble after cleavage by cathepsin S and activates microglia, causing IL-1β release and central sensitization [50].

Chemokines also regulate the movement of immune cells and provide communication between neural cells and non-neural cells [49]. CCL2 and its receptor CCR2 regulate monocyte and macrophage movement to injured nerves and the DRG. CXCL1 can act on CXCR2-expressing neurons and cause hyperexcitability of the nerve cells [49]. After being released from the cell membrane, neuronal CX3CL1 can signal to CX3CR1-expressing microglia [49,50]. The numerous cytokines involved in the pathogenesis of neuropathic pain form a complex signaling network, making this system a potential pharmacological target for the treatment of neuropathic pain. CC chemokine receptor antagonism and combined CCR2/CCR5 blockers can produce an anti-inflammatory and antinociceptive effect in preclinical studies [51].

CX3CL1 is a potent chemoattractant for B cells, T cells, NK cells, and monocytes. The receptor for CX3CL1 (fractalkine) is found to be significantly upregulated by microgliosis induced by nerve injury. CX3CL1/CX3CR1 is an important pathway of communication between neurons and microglia during neuropathic pain states [52].

CX3CL1 is primarily found on sensory neurons and dorsal horn neurons and is anchored to the cell membrane by a mucin stalk [53]. This membrane-bound CX3CL1 can be cleaved by protease activity. When primary afferent stimulation occurs, lysosomal cysteine protease cathepsin S (CatS) is released from microglia and cleaves membrane-bound CX3CL1. ATP causes the release of BDNF from microglial cells by activating P2X4R. When BDNF is released, it binds TrkB receptors on neurons, inducing a shift in chloride anion gradient in dorsal horn nociceptive neurons. This induces the excitability of these nociceptive neurons through GABAA receptor-mediated depolarization [53].

Studies have found a marked upregulation in CSF1 and colony-stimulating factor 1 receptor (CSF1R) in the ipsilateral dorsal cord after nerve injury [54]. Induction of CSF1 in both small- and large-diameter DRG neurons occurred within 18 h of injury and persisted for 3 weeks. CSF1 was not detected in the absence of injury. The upregulation of CSF1 in injured sensory neurons has been found to trigger neuropathic pain through the activation of microglial cells and relevant microglial genes. Additionally, CSF1 was found to transform resident microglia to a highly proliferative, self-renewal state of the microglia [54].

### 4.3. Complement Activation in Nerve Injury

The complement system is an important part of the immune system and is activated after peripheral nerve injury through the classical, lectin, and alternative pathways [55]. The activation of the complement system creates opsonins and anaphylatoxins like C3a and C5a. These processes increase the neural inflammation and sensitivity of the nociceptor. Therefore, the complement system is directly linked to pain development [55].

The complement system forms the membrane attack complex (MAC) at the end of the cascade, which causes cell injury and neuroinflammation in nerves and Schwann cell membranes [55]. Therefore, treatment targeting the complement system is an important investigation area since uncontrolled activation of the complement system contributes to several neurological diseases.

### 4.4. Neuroimmune Signaling and Resolution in Chronic Pain

Acute inflammation normally facilitates the removal of damaged material and supports tissue repair; however, when the inflammatory response fails to resolve, the production of cytokines and chemokines continues, maintaining the activation of immune cells and glial cells. Additionally, sensitized neurons may further continue inflammation [56]. These processes lead to the original injury becoming less important than the subsequent feedback loops [56].

Anti-inflammatory and pro-resolving mediators help to maintain homeostasis after inflammation [56]. Therefore, resolution is an active process rather than simply the disappearance of inflammation. If these mechanisms fail, this can cause persistent immune activation and nociceptor sensitization and lead to chronic pain [56].

In the early stages of neuroinflammation, activated signaling pathways like nerve growth factor (NGF)/TRPV1 signaling and specificity protein 4 (Sp4)-related transcriptional regulation can cause long-lasting changes in sensory neurons. On a molecular basis, these changes can transform normal nociceptor signal to a pathological chronic peripheral sensitization [57].

Chronic pain after a peripheral nerve injury is not caused only by neurons but also by the interactions of several cell types like infiltrating macrophages, Schwann cells, microglia, astrocytes, and sensory neurons. These cells can influence one another at the level of the injured nerve, the DRG, the spinal cord, and higher levels of the nervous system [33]. This may explain the reason for persistent pain even after the initial injury. For successful treatment, a better understanding of these interactions and approaches that target persistent neuroimmune signaling rather than simply relieving the symptoms is needed [33,56,57].

## 5. Biomarkers and Molecular Targets for Immune Modulation in Chronic Pain

### 5.1. CD55 and Complement Regulation in Neuroinflammation

CD55, also known as decay-accelerating factor (DAF), is a cell surface protein that protects against complement attack. CD55 regulates the activation of complement by accelerating decay of C3 and C5 convertase [58]. CD55 is linked to the cell membrane via a glycosylphosphatidylinositol (GPI) anchor [58]. This inhibitor is especially helpful when protecting healthy tissue near an injured area with active complement signaling, as in cases of nerve damage [55]. When this inhibitor is not regulated properly, it leaves healthy cells susceptible to complement deposition. When complement deposition occurs in healthy tissue as a result of recruitment to a site of injury, this can yield neuropathic pain [55].

Some pain studies have shown a significant decrease in CD55 in high pain states and has been used to predict high pain states in patients [59]. CD55 has different behaviors regarding different types of nerve injury. In a crush injury, CD55 decreases immediately after injury and is recovered consistently with the healing of injury [55]. In chronic transection, the levels of CD55 never return to baseline, indicating that injury is not resolved and complement activity is chronically disinhibited [55].

### 5.2. TNF Signaling as a Target of Immune Modulation

TNF-α is a major pro-inflammatory cytokine that is released in response to injury and inflammation. TNF-α binds to two different receptors, TNFR1 and tumor necrosis factor receptor 2 (TNFR2). TNFR1 is present on all human tissue cells, while TNFR2 is only found on endothelial cells, immune cells, and neurons [46]. Both of their intracellular domains are also structurally and functionally distinct because they trigger different signaling cascades [60].

TNFR1 is the main regulator of immune responses, complement activation, cytokine release, and neutrophil migration. Many of the specific pathways that TNFR1 triggers lead to the activation of transcription factors and mediate pro-apoptotic molecules [60]. Conversely, TNFR2 is mainly linked to cell survival and immune regulation rather than apoptosis. In other words, TNFR1 is mainly responsible for promoting inflammation and tissue destruction, while TNFR2 assists in maintaining a state of homeostasis through cell survival and tissue regeneration [60].

TNF-α is a crucial component in neuroinflammatory processes associated with peripheral and central sensitization [61]. TNF-α upregulates voltage-gated sodium channels (VGSCs) that play a central role in nociceptive transmission in the DRG, increasing the neuronal excitability and generating more action potentials in primary afferent neurons [61]. This process produces peripheral sensitization and increases the intensity of pain signaling, causing mechanical allodynia and hyperalgesia. Regarding central sensitization, TNF-α enhances excitatory postsynaptic currents (EPSCs) in the spinal cord through AMPA and NMDA receptors. This potentiation of excitatory signals determines pain signaling amplification and central sensitization in cases of chronic pain [61].

Regarding therapeutic solutions, TNF-α inhibitors have been proposed and have shown promise, but there have been setbacks because these drugs come with a ‘black box’ warning due to their adverse effects, including reactivation of latent tuberculosis, opportunistic infections, lupus-like symptoms, sarcoidosis, and an increased potential for development of lymphoma [60]. TNF and CD55 and their key roles are seen in Table 1.

### 5.3. Emerging Immune Biomarkers in Chronic Pain

#### 5.3.1. Complement Components

The biomarkers discussed below should be interpreted according to the biological process they reflect and their level of clinical validation. Some include candidate markers of systemic or neuroinflammatory activity, whereas others reflect nerve injury. Most remain exploratory and are not validated for routine diagnoses. Elevated levels of C3 and C5 are mostly attributed to their production by glial cells. They often form their soluble fragments, C3a and C5a, by their convertases, and some studies have shown significant elevation of C3a and C5a in patients with acute and chronic pain conditions [55].

#### 5.3.2. HMGB1-Mediated Damage Signaling

HMGB1 is an important component in neurogenic inflammation. HMGB1 is expressed in cortical neurons and nociceptors. The neuronal expression of HMGB1 is enhanced during nerve and tissue injury [62]. HMGB1 is mainly secreted by microglial cells, which are the primary innate immune cells in the CNS. Microglia express TLRs, which control the pro-inflammatory effects of HMGB1 in peripheral innate immune cells [63]. HMGB1 has many receptor interactions: RAGE, TLR9, TLR4, TLR2, CD24, and CXCR4. When HMGB1 binds to TLR4 and RAGE on the cell surface, this triggers the transcription of inflammatory cytokines such as IL-1β, IL-6, and TNF-α [64].

HMGB1 induced neuroinflammation and necrosis after injury through its interaction with RAGE and TLR4. HMGB1’s role in neuroinflammation has been confirmed with HMGB1 blocking studies that have demonstrated significant neuroprotection [63]. HMGB1 binds lipopolysaccharides (LPS) and IL-1 to prompt TLR4-mediated pro-inflammatory responses. The release of HMGB1 is dependent on different processes such as phosphorylation by calcium-dependent protein kinase C. HMGB1 has been implicated in many neuroinflammatory-related pathologies including epilepsy, multiple sclerosis, and Alzheimer’s disease [63].

#### 5.3.3. Neurotrophic Factors and Neural Plasticity

NGF plays a key role in the regulation of peripheral innervation [65]. Neurotrophins such as NGF and BDNF bind to Trk receptors. NGF binds to TrkA, and BDNF binds to TrkB, which leads to the intracellular activation of signaling pathways. BDNF/TrkB is necessary for the survival of CNS neurons and plays a critical role in neuronal plasticity [66].

#### 5.3.4. Biomarker-Guided Precision Medicine

Inflammatory biomarkers have shown promising results in quantifying glial activation at relevant anatomical sites. For example, translocator protein 18 kDa (TSPO) imaging studies have shown a significant elevation in immune cell density in the skull bone marrow of patients with chronic lower back pain and osteoarthritis [13]. This opens a new avenue regarding the interface between CNS and PNS in chronic pain, providing potential therapeutic targeting. On a separate note, the use of the most studied cytokines, TNF, IL-1β, IL-6, IL-8, CCL2, BDNF, or NGF, lacks consistency. For instance, CSF cytokines in chronic pain show dysregulation, but correlations with pain severity are not consistent [13].

CSF-1R signaling has been investigated primarily as a therapeutic target for modulating myeloid-cell activity rather than as an established biomarker. Pexidartinib has been used for the treatment of tenosynovial giant cell tumors, and it showed physical function and pain improvements, although this therapy requires monitoring for hepatotoxicity and microglial depletion [13]. Although evidence for pexidartinib comes from giant cell tumors rather than chronic pain, its effect on the CSF pathway provides exploratory evidence for the therapeutic relevance of this target.

## 6. Recovery Mechanisms and Resolution of Neuroinflammation

### 6.1. Active Resolution of Inflammation

Neuroinflammation is a very dynamic and context-dependent process. Many factors, including aging, protein aggregates, and cell stress, could potentially influence neuroinflammatory responses [67]. Thus, inflammation is an active process, as it is tightly regulated to restore tissue homeostasis. Chemokines are one of the main promoters of this process, recruiting T-regs, generating autoantibodies, and inducing tissue repair [68]. Additionally, specialized pro-resolving mediators (SPMs) are broad and regulate macrophage activity during inflammation and resolution, illustrating the interconnectedness between immune cells [69]. In diseases like Alzheimer’s and Parkinson’s, SPMs have been shown to alter the expression of pro-inflammatory genes and promote the resolution of neuroinflammation. These findings provide mechanistic insight into the resolution of neuroinflammation but are not direct evidence for chronic pain. A better understanding of SPMs could potentially improve Alzheimer’s and Parkinson’s treatment [70].

### 6.2. Immune Cell Reprogramming

Evidence from related inflammatory and tissue-repair conditions is included here when it provides mechanistic insight into immune-cell regulation and resolution processes relevant to chronic pain, as these findings are complementary. Tissue repair has multiple phases, with the early phase being dominated by pro-inflammatory macrophages, often described as M1-like, with subsequent changes toward reparative M2-like phenotypes. Macrophage activation exists along a spectrum and is highly context-dependent rather than representing a simple M1-to-M2 transition. In a study observing macrophage interaction with IL-4, it was shown that polarization can promote changes toward a M2 phenotype, leading to changes in gene expression and functional differences [71]. Furthermore, in a study of rheumatoid arthritis, the apoptosis of M1 macrophages and repolarization of M1 to M2 helped repair joint damage through nano-enzymes [72]. This evidence comes from an inflammatory joint-disease model and should be considered mechanistic support rather than direct evidence for chronic pain treatment. These findings suggest that macrophage reprogramming may contribute to resolution, but recovery cannot be explained by a simple binary M1 to M2. This pathway also shows how the M1 macrophages’ overabundant release of inflammatory cytokines could be reversed through reprogramming [72]. T-regs have been shown, when delivered exogenously, in injured bone, tissue, and skin, to promote tissue repair and enhance healing. Although these findings demonstrate effects on tissue repairs, their relevance to chronic pain remains indirect. They accomplish this by modulating both monocytes and macrophages at the site of injury to produce pro-healing factors like IL-10 [73]. Also, in cancer cells, it has been shown that reprogramming T-regs can reshape the tumor and promote regression [74]. These findings provide indirect mechanistic support for T-reg-mediated immune regulation but do not directly establish an effect on chronic pain.

### 6.3. Peripheral Nerve Regeneration

When nerves are injured, Schwann cells are reprogrammed through the downregulation of myelin genes combined with the activation of repair-supportive features. The repair-supportive features could include the elevation of cytokines, myelin autophagy, and regenerative tract formation [75].

Axonal regeneration can also promote peripheral nerve regeneration, and this can be accomplished through the release of growth-promoting neurotrophic factors. Spinal cord stimulation is a way to use electrical conductivity to help induce this process and improve neurotrophic factor release [76]. The restoration of normal peripheral nerve function is a growing field of research. One method, nerve guidance conduits, has shown early promise as they simulate artificial channels to guide regenerating nerves [77]. The ultimate goal of restoring nerve function is to enable increased plasticity, and upregulating brain stimulation is the key to this occurring [78]. A summary of the mechanisms of neuroinflammation is seen in Table 2.

## 7. Current and Emerging Immunomodulatory Therapies

Conventional anti-inflammatory therapies have shown inconsistent effects in preventing the development of chronic pain. A meta-analysis of 29 randomized trials including 5220 patients found no evidence to support the use of NSAIDs or corticosteroids for the prevention of chronic nociceptive pain [79]. Corticosteroids were associated with a 53% lower overall rate of chronic neuropathic pain when given during the acute phase (risk ratio (RR) 0.47, 95% confidence interval (CI) 0.36–0.62), but the evidence was low quality and the studies differed considerably from one another [79]. Studies of NSAIDs and corticosteroids have produced mixed results when researchers looked at whether acute pain later became chronic. This has led to more interest in the individual inflammatory signals involved in pain.

TNF-α, IL-1, and IL-6 have each been investigated as potential therapeutic targets in neuropathic pain. Clinical results with TNF-α inhibitors have been mixed. Epidural etanercept provided better short-term relief of radicular leg pain than dexamethasone [80], whereas intravenous infliximab was not better than placebo for sciatica associated with disc herniation [81]. Animal studies suggest that IL-1 and IL-6 signaling may be useful treatment targets, but there is still limited evidence showing a benefit in humans [82,83]. A small, uncontrolled study reported improvement following tocilizumab treatment, but its design limits conclusions regarding efficacy [84].

The glial response after nerve injury has also been studied as a possible treatment target. Minocycline has made it into human trials, but the results have been inconclusive. In one study, patients with lumbar radicular pain improved slightly, although the difference was too small to be considered clinically meaningful [85]. Patients who received minocycline around the time of lumbar discectomy did not have less persistent pain than the control group [86]. In a rat model of nerve injury, duloxetine reduced mechanical allodynia and also decreased P2X4 receptor activity in microglia, suggesting that this pathway could play a role in its analgesic effect [87]. Sensory-neuron-derived CSF1 was also necessary for microglial activation and mechanical hypersensitivity following nerve injury in mice [54], while pharmacologic CSF1R inhibition reduced microglial and macrophage activation and both prevented and reversed allodynia in a separate mouse model [88].

The complement system has also emerged as a potential target for treating neuropathic pain and promoting recovery after peripheral nerve injury. In a rodent nerve-injury model, the inhibition of the C5a receptor reduced cold allodynia, supporting C5a signaling as a potential therapeutic target [89]. Complement inhibition may also promote nerve recovery, as treatment with soluble complement receptor 1 accelerated sensory recovery and nerve regeneration following sciatic nerve injury in rats [90]. Endogenous complement regulators may provide another approach. Local treatment with the complement regulator CD59 reduced mechanical allodynia and membrane attack complex deposition following peripheral nerve injury [91]. Although CD55 was not tested therapeutically, its gene and protein expression decreased following nerve injury [91].

Advanced biological therapies provide another approach for targeting pathways involved in neuropathic pain. A phase II study of the anti-NGF antibody fulranumab found greater pain reduction at higher doses in patients with diabetic neuropathic pain, but the trial ended early, which limited the strength of the findings [92]. VM202, a plasmid encoding hepatocyte growth factor, was associated with significant pain improvement in an earlier placebo-controlled trial of diabetic neuropathy [93], but a subsequent 500-patient phase III trial failed to meet its efficacy endpoints, although a smaller extension cohort showed improvement [94]. RNA-based therapies remain preclinical, with microglial-targeted delivery of small interfering RNA (siRNA) against interferon regulatory factor 5 (IRF5) reducing mechanical hypersensitivity in a mouse model of neuropathic pain [95].

Stem cell-derived exosomes are another area of interest because they may reduce inflammation while also helping injured nerves recover. In one rat study, treatment with exosomes from human umbilical cord mesenchymal stem cells reduced mechanical allodynia and reduced inflammatory activity in spinal microglia [78]. In a systematic review of preclinical studies, exosome-containing scaffolds were generally associated with better functional recovery and nerve regeneration than scaffolds alone, although the studies used a wide range of methods [96]. Macrophage-derived extracellular vesicles may also be important, as reducing micro RNA-155 (miR-155) signaling through these vesicles attenuated mechanical hypersensitivity in a preclinical model [97]. These findings are encouraging, but further studies are needed before these approaches can be used clinically.

Since neuropathic pain can be driven by more than one mechanism, treating several pathways at the same time may be more useful than focusing on only one. In practice, more than one medication is often needed when a single agent does not provide adequate pain relief [98]. Preclinical work also supports treating more than one pathway at a time. In a rat model, the combined gene therapy produced greater pain relief than approaches using fewer components [99]. In the future, treatment may also be guided by the specific mechanisms or biomarkers present in each patient, although reliable biomarkers for selecting neuropathic pain therapies are not yet well established [98].

## 8. Challenges and Future Directions

### 8.1. Translational Challenges in Neuroimmune Pain Research

Part of the challenge in researching neuropathogenesis is due to the lack of reliable animal models [100]. These models are known as experimental autoimmune neuritis (EAN) and they act, for example, as models for Guillain–Barre Syndrome (GBS) by inducing peripheral nerve demyelination, through the use of PNS myelin proteins [101].

The most reliable of these EANs is the Lewis rat model, due to its close resemblance to human GBS; however, identifying useful targets for treatments using this model is still challenging because many strains of rat are resistant to the necessary gene knockouts [100]. When it comes to research on neuropathogenesis, until recently, there have not been many reliable informative biomarkers that could be used for early detection [102]. Only recently has there been an investigation into the mechanisms behind autoimmune diseases and locating more specific biomarkers [103].

### 8.2. Advances in Diagnostic and Biomarker Discovery

Typically, the methods for identifying many types of immune-mediated neuropathies include some form of neuroimaging [103,104]. Electrophysiological testing can identify abnormalities associated with nerve damage, such as increased conduction time, prolonged or repeated F-waves (late motor responses), and an increase in A-waves (abnormal recurrent motor responses) [105]. However, while this method is an effective tool for the identification of nerve damage, it is an indirect measure, provides little information regarding the pathogenesis of injury, and is nonspecific to the type of nerve damage at the site [103].

Another standard method is quantitative sensory testing (QST), which assesses the overall sensitivity of the nervous system. If the patient shows abnormally reduced sensitivity, then it could be an early indicator of peripheral nervous damage. Even though this method of identification is considered one of the gold standards of diagnosis for diabetic peripheral neuropathy (DPN) [106,107], it has trouble detecting damage in smaller peripheral axons.

Because of the issues concerning traditional biomarkers, there has been more research on desirable biomarkers. A desirable biomarker would be more direct, specific, sensitive, have high ease of use, and would be more cost-effective [103]. One of those potential biomarkers is sphingomyelin, a lipid that mainly constitutes myelin sheaths [108]. Myelin sheaths in the PNS are created by Schwann cells that surround the axons of the longer neurons, and act as insulators that help speed up electrical conductions [109,110].

In the event of immune cell infiltration, the damage could result in the degradation and unraveling of the myelin sheaths, so the unique components of their membranes are hypothesized to be an indicator of neuroinflammation and demyelination [108,109,110]. Although these biomarkers are investigated primarily as indicators of peripheral nerve demyelination, rather than pain itself, they may provide exploratory information relevant to immune-mediated neuropathic pain. Levels in the CSF were found to be significantly higher in patients diagnosed with chronic inflammatory demyelinating polyradiculoneuropathy (CIDP) and GBS [102]. Additionally, the levels of sphingomyelin that were detected increased, which was consistent with clinical scales including the Inflammatory Neuropathy Cause And Treatment (INCAT) scale and the Overall Neuropathy Limitations Scale (ONLS), which rank the severity of CIDP and GBS [108]. For peripheral nerve demyelination specifically, plasma periaxin serum concentrations could serve as a potential biomarker in cases like CIDP and GBS [103]. It was determined that periaxin, unlike other indicators like neurofilament light chains or sphingomyelin, is specifically expressed by myelinating Schwann cells of the PNS [103]. Experimental trials showing low levels prior to immune-mediated demyelination, followed by high levels of expression afterward only in the PNS tissue samples, demonstrate its potential use as a marker [103].

### 8.3. Future Directions in Immune-Based Pain Therapy

The pain caused by immune-mediated nerve damage can often be caused by excessive inflammation induced by the immune system [111]. The complement pathway usually induces a controlled amount of inflammation that is required for the repair of demyelinated or damages nerves [112]. However, if inflammation persists, it can lead to perpetual demyelination and further nerve damage [40]. Some methods of treatment would involve the precise regulation of the immune system via the inhibition of NLRP3 inflammasomes, which may shift macrophage activity away from sustained inflammatory states toward reparative and resolution-associated functions [111].

Other components of the complement system that are targets for pain relief include B-lymphocytes, which generally increase peripheral sensitivity through the secretion of autoantibodies [40]. T cells also directly and indirectly affect pain by either releasing IL-10 by CD4+ T cells, which is also part of the process for modulating macrophage inflammatory and reparative functions [29], or through the production of certain opioid peptides, such as enkephalins, which can be regulated by exogenous opioid peptides [40]. To avoid these outcomes, these therapies specifically attempt to rebalance the immune system by precisely controlling only the overexpressed parts of the immune system.

## 9. Conclusions

Neuroimmune interactions are important in the development and the persistence of chronic pain. Chronic pain can occur following tissue injury and peripheral nerve injury, and the persistent activation of immune cells, nociceptors, microglia, and astrocytes can sustain the inflammatory response, inflammatory signaling, and central sensitization.

Cytokine, chemokine, and complement pathways are all examples of how molecular targets can be used as immune-based therapies. Biomarker research also may be used to distinguish the different pain mechanisms, but reliable biomarkers for diagnosis and treatment selection still need to be established. Recovery depends on suppressing inflammation and on active resolution, immune cell reprogramming, and peripheral nerve regeneration. Overall, the immune system plays an important role in chronic pain, and targeting different immune pathways can be used in pain management. While neuroimmune pathways provide strong mechanistic and preclinical rationale for therapeutic development, clinical efficacy remains variable, highlighting the need for rigorous translation from experimental findings to validated treatments.

## Figures and Tables

**Table 1:** CD55 and TNF-α and their targets and roles in immune nodulation of chronic pain.

Molecular Target	Primary Effect	Role in Neuroinflammation and Chronic Pain	Clinical Relevance
TNF-α	Pro-inflammatory [46]	Increases neuronal excitability through upregulation of voltage-gated sodium channels which promote peripheral and central sensitization [61]	TNF-α inhibitors can be used as therapeutic approaches [60]
CD55 (DAF)	Complement-Regulatory [55]	Regulates complement activation by accelerating C3/C5 convertase decay, which limits complement-mediated tissue injury [58]	Biomarker and target for complement regulation [55]

**Table 2:** Key roles and mechanisms in neurohumoral and pain responses.

Mechanism	Key Role	Effect on Inflammation and Repair
Active Resolution of Inflammation	Chemokines recruit T-regs and promote tissue repair [67]	Decreases inflammation and increases resolution [68]
Immune Cell Programming	Macrophage activation shifts along a context-dependent spectrum [71]	Decreases pro-inflammatory activity and increases tissue repair [71]
Peripheral Nerve Regeneration	Schwann cell reprogramming supports nerve repair [75]	Increases nerve repair and functional recovery [76]

## Data Availability

No new data were created or analyzed in this study. Data sharing is not applicable to this article.

## Abbreviations

ATP	adenosine triphosphate
BDNF	brain-derived neurotrophic factor
BNB	blood–nerve barrier
CatS	cathepsin S
CCR2	C-C chemokine receptor 2
CGRP	calcitonin gene-related peptide
CI	confidence interval
CIDP	chronic inflammatory demyelinating polyradiculoneuropathy
CNS	central nervous system
CSF1	colony-stimulating factor-1
CSF1R	colony-stimulating factor 1 receptor
CX3CL1	C-X3-C motif chemokine ligand 1
CX3CR1	C-X3-C motif chemokine receptor 1
CXCL1	C-X-C motif chemokine ligand 1
CXCR2	C-X-C motif chemokine receptor 2
DAF	decay-accelerating factor
DAMPs	danger-associated molecular patterns
DPN	diabetic peripheral neuropathy
DRG	dorsal root ganglia
EAN	experimental autoimmune neuritis
EPSCs	excitatory postsynaptic currents
FOXP3	forkhead box p3
GABA	gamma-aminobutyric acid
GBS	Guillain–Barre syndrome
GLT1	glutamate transporter 1
GPCRs	G-protein coupled receptors
GPI	glycosyl phosphatidylinositol
HMGB1	high-mobility group box 1
IASP	International Association for the Study of Pain
ICD-11	International Classification of Diseases, 11th Revision
ICU	intensive care unit
IL-1	interleukin-1
IL-1β	interleukin-1β
IL-6	interleukin-6
IL-10	interleukin-10
IL_17	interleukin-10
INCAT	Inflammatory Neuropathy Cause and Treatment
IRF5	interferon regulatory factor 5
LPS	lipopolysaccharides
MAC	membrane attack complex
MAPK	mitogen-activated protein kinase
MCP-1	monocyte chemoattractant protein-1
miR-155	microRNA-155
MRGPRB2	mas-related G protein-coupled receptor member B2
MRGPRX2	mas-related G protein-coupled receptor member X2
NF-κB	nuclear factor-κB
NGF	nerve growth factor
NLRP3	nucleotide-binding domain, leucine-rich-containing family, pyrin domain-containing-3
NMDA	N-methyl-D-aspartate
NSAIDs	nonsteroidal anti-inflammatory drugs
ONLS	Overall Neuropathy Limitations Scale
PNS	peripheral nervous system
PRRs	pattern-recognition receptors
QST	quantitative sensory testing
RAGE	receptor for advanced glycation end products
ROS	reactive oxygen species
RR	relative risk
siRNA	small interfering RNA
Sp4	specificity protein 4
SPMs	specialized pro-resolving mediators
STAT3	signal transducer and activator of transcription 3
TLRs	Toll-like receptors
TNFR1	tumor necrosis factor receptor 1
TNFR2	tumor necrosis factor receptor 2
TNF-α	tumor necrosis factor-α
TRPA1	transient receptor potential ankyrin 1
TRPV1	transient receptor potential vanilloid 1
TSPO	translocator protein 18 kDa
VGSCs	voltage-gated sodium channels
IBA-1	ionized calcium-binding adapter molecule 1
